# Military surgery as national romance: the memory of British heroic fortitude at Waterloo

**DOI:** 10.1080/07292473.2020.1741771

**Published:** 2020-04-21

**Authors:** James Kennaway

**Affiliations:** University of Roehampton, Roehampton, UK

**Keywords:** Waterloo, surgery, national romance, romantic militarism, heroism

## Abstract

This paper shows the ways that tales of stoicism during surgery at the Battle of Waterloo came to be a significant part of the ideological framework of Romantic Militarism. Celebrating the killing of enemies clashed with ideals of politeness, but hailing a soldier’s powers of endurance in surgery was an acceptable way of extolling courage, framing lived experience of agony into narratives of exalted pain, masculine fortitude and quasi-religious patriotic feeling. In Britain, an extensive discourse emerged about the supposed Britishness of surgical sangfroid at Waterloo, providing a narrative of national superiority in the decades of imperial expansion that followed.

Stendhal’s 1830 novel *The Red and the Black* is often mentioned as a dissection of the impact of the memory of Napoleonic glory on ambitious young men in the more banal decades that followed Waterloo. The author (real name Marie-Henri Beyle), who had been on campaign with Napoleon, depicts his protagonist Julien Sorel trying to rise in society and living out his petty adulteries in an ironic sub-Napoleonic key. It is less well known, however, that Sorel’s dreams of martial destiny are derived not from a soldier but from ‘an old Surgeon-Major of Napoleon’s Italian Army’, who provided him with the early education in Latin and in ‘the battles of Lodi, Areola and Rivoli’ that allows Sorel to escape from provincial obscurity.^1^ Thereafter, his young mind is dominated by a secret ‘insane passion’ both for Napoleon and for ‘the circumstantial details of the most terrible [surgical] operations’.^2^ When his first mistress tries to engage him in conversation, Sorel replies with ‘talk of surgical operations’, out of ignorance of other topics.^3^ His fascination with the details of battlefield surgery is not dry or academic but related to a romance of sublime pain, of manly self-sacrifice as a rejection of shallow bourgeois values. Naturally, he swears to himself that he would ‘not have flinched’ under the knife.^4^

With this view of battlefield surgery, Julien Sorel was in many ways a representative figure. In the wake of the Napoleonic Wars, tales of stoicism during (unanaesthetised) surgery came to be a standard part of the developing cultural and ideological framework of what Nancy Rosenblum has called Romantic Militarism.^5^ By 1815, its conception of war as a heroic means to achieve justice, liberty and manly self-expression had given combat a new glamour as something big, important and even spiritual compared to the humdrum world of making a living.^6^ In this context, openly celebrating the actual killing of enemies may still have clashed with ideals of politeness and Christian charity, but hailing a soldier’s powers of endurance in surgery was an acceptable way of extolling courage, framing lived experience of agony into narratives of exalted pain, masculine fortitude and quasi-religious patriotic feeling.^7^ As such, it involved a vision of war wounds as an escape from alienation and bourgeois values that had much in common with another well-known aspect of Romanticism – the obsession with suicide. Both rejected society in a cult of egoistic self-sacrifice. It was an anti-pastoral, offering an ideal outside normal life, not of the shepherd but of the martyred warrior.

In Britain this Romantic cult of heroic bravery in surgery often took an explicitly national and patriotic form, with a widespread debate on the supposed Britishness of surgical sangfroid.^8^ Endlessly retold in many different genres, such stories helped to forge a sense of British identity, providing a comforting narrative of national difference and superiority in the decades of imperial expansion that followed.^9^ They were seen as somehow British in spirit, perfectly suited to a conception of the nation as emotionally restrained, virile and brave.^10^ Anecdotes of fortitude in surgery entered the canon of tales of natural pluck, adversity overcome and victory against the odds in a fully-fledged ‘National Romance’, the providential triumphs of a free Protestant country.^11^ These stories, whether related to officer-class nonchalance or rough-and-ready machismo from other ranks, seemed to imply a natural patriotic bond above class distinctions that was apparently irresistible. As such, they were grist to the mill of the developing culture of popular militarism, reflecting the fact that, far from being an exception to the militarism prevalent on the continent, as national mythology would have it, Britain was if anything a front-runner.^12^

The most prominent battle in this discourse of heroic British surgical fortitude was Waterloo, an unusually sanguinary encounter that was quickly understood to mark the end of the long struggle with France. As the final cataclysm of the war, the battle was a vital obsession in nineteenth-century Romantic Militarism, a last hurrah of glory before the lacklustre years of peace. Strategic and tactical concerns meant that both the British (or rather Anglo-German-Dutch) forces and their French opponents remained on the field despite heavy losses. By the time the French army collapsed on the evening of 18 June, perhaps 50,000 men were left dead or wounded.^13^ This made the battle, and especially its aftermath, a very large-scale surgical affair indeed, with a huge number of amputations, several involving what one might call celebrity amputees. Along with British squares stolidly holding out and the pathos of deaths such as that of William de Lancey, narratives of heroic composure in surgery became an important aspect of the memory of the engagement. Admiration of courage in military surgery reflected a masculine and soldierly culture of self-respect and fortitude that, in different forms, doubtless stretches back throughout military history. However, the mediation of such experiences created a substantial cult of Waterloo, offering, as Philip Shaw has noted, a touch of the sublime – a sublime not of nature, but of human death, sacrifice, pain, endurance and patriotic fervour^14^

In many ways, this ‘official’ discourse of heroic British stoicism in surgery might seem a bad joke, so hyperbolic as to make one think of it as pure ideology, a fantasy cut from whole cloth with no relation whatever to what went on in the real world. It is certainly the case that depictions of wounded men often showed, in Shaw’s words, a ‘disregard for the inner experience’.^15^ It is hard to believe that amputations and other unanaesthetised operations at Waterloo were not generally accompanied by despair, blind panic and overwhelming pain, and plenty of sources reflect that. For instance, Henry Addison, formerly of the Life Guards, wrote that it ‘it was almost impossible to sleep’ after the battle because of ‘the frequent sharp cry of agony as the surgeon’s knife performed some necessary amputation’.^16^ Similarly, William Hay, an officer in the 12th Light Dragoons, said that the sight of surgery after Waterloo was worse than battle and made him ‘feel quite sick’.^17^

Nonetheless, it seems that the actual experience of military surgery was sometimes also powerfully mediated by ideas of masculine self-control and patriotism that gave meaning to the subjective experience of agony and dismemberment. While there were clearly some, like Stendhal’s callow hero Fabrice in *The Charterhouse of Parma*, wounded by his own side at Waterloo, who were ‘liberated’ from ‘the romantic element’ of war by the experience of injury, eyewitness testimony of extraordinary bravery in surgery of the kind described in this article was so common that one has to take it seriously.^18^ One should not draw too sharp a dichotomy between experience and later mediation, since testimony about the lived experiences of the men present was also mediated by ideologies of patriotic manly endurance. An anonymous observer of the battle, writing in his *Flying Sketches of the Battle of Waterloo*, noted that, ‘the wounded men seem to vie with each other in bearing their agonies with fortitude; and we actually saw two British soldiers disputing the precedence of valour at some particular moment, one of whom had lost a leg, and the other was severely wounded in the arm!’^19^ Such descriptions have little of the glamour, dubious humour or heavy-handed ideology of later myth-making, but they nevertheless suggest the potential power of ideals of toughness, courage and manliness in framing the actuality of surgery.

First-hand testimony on the reality of Waterloo surgery from the many British surgeons who went to Brussels in the wake of the battle also reflected both the horrors involved and admiration for soldiers’ fortitude, often expressing profound sympathy in a way that undermines the clichés of the surgeon of the period as callous.^20^ Charles Bell spoke of ‘the most shocking sights of woe, to my ear accents of entreaty, outcry from the manly breast, interrupted forcible expressions of the dying, and noisome smells’, he had to witness, which left him with a ‘gloomy, uncomfortable view of human nature’ and sceptical of the ‘gallant stories, the charges, the individual instances of enterprise and valour’ that the world associated with ‘victory and Waterloo’.^21^ However, he also acknowledged the fortitude of the men, writing that ‘there was no expression of suffering’.^22^ The famous paintings of his patients that Bell produced after Waterloo also reflected not only what Shaw has called an ‘unsettling pathos’ but also a sober appreciation of men’s courage.^23^

After the battle, the experience of surgery was further mediated into a widespread cultural memory of British fortitude in surgery at Waterloo that had a significant impact.^24^ Within days of the battle, newspapers were putting out reports of British courage during surgery. Innumerable subsequent memoirs, journals and medical texts supplied further examples, many of which were simultaneously embellished and boiled down to the level of a patriotic anecdote, entering the repertoire of stirring tales of British courage for the National Romance. In this way, heroic British fortitude in surgery was firmly established as a semi-mythical subgenre. It evoked a scene of individual Homeric heroism more adapted to ideals of Romantic Militarism than the iron discipline needed to maintain a square under fire, transfiguring the trauma and virile courage of the reality of surgery at Waterloo into a story of latter-day knights sacrificing themselves for a higher calling. Focusing on surgical endurance provided a way of glorying in the triumph of British arms that did not dwell on the brutality of the infliction of bloodshed, which could provoke guilt and which clashed with the country’s sense of itself as a polite and Enlightened nation marked by sensibility. Likewise, in the context of the emerging evangelical revival, by stressing the capacity for enduring pain rather than for imposing it, those memories also helped cast a veil on the ways that Britain’s might (like that of other states) depended on an ability to mete out violence, maintaining an image of Britain as an essentially moral Christian power.

This article considers the experience of surgery at Waterloo and the ways it was remembered as a field of manly heroic endeavour, sublime agony and national valour. First, it considers the notion that there was something particularly British about such heroism in in the context of the British National Romance, before turning to issues of how that related to the class divisions within British society. It is hoped that it represents a step towards a view of the history of nineteenth-century surgery that goes beyond progress narratives of great surgeons, to focus on the attitudes of patients and other observers. Likewise, previous historiography has arguably somewhat neglected military surgery in favour of its civilian counterpart, although the army and navy accounted for a significant percentage of all serious surgical operations.^25^ Many civilian surgeons rarely conducted actual operations at all, and, famously, many flocked to Waterloo to get experience with ‘capital operations’ such as amputations that they could never get at home. In the wake of the Napoleonic Wars, battlefield surgery loomed large in cultural understandings of what surgery in general meant. Seen in this light, these Napoleonic-era stories of manly fortitude under the knife are not a marginal topic but one with real significance for thinking on surgery, war, masculinity and nationhood.

## National character in Waterloo surgery

Victory at Waterloo, including stories the apparent fortitude of British men in surgery, was balm to elite anxieties about national character. Defeat in the American War of Independence and inept interventions on the continent in the 1790s had led to fears that British commercial success was leading to luxury and a weakening of exactly the sort of martial resolve necessary for composure during surgery.^26^ Eventual victory appeared to trump that argument for a generation, even if final success was more to do with the sinews of the fiscal-military state than with the native manly virtues of an army that was often fairly international in any case. Nevertheless, Britons could revel in their warlike and imperturbable reputation in a new way. In an 1816 article, Walter Scott expressed great satisfaction that the foreigners he encountered entertained ‘high ideas’ about:

the steadiness, valour, energy and discipline of the British army. It was remarked to me, that scarcely any other troops possessed that firmness and discipline, joined to what we would call *bottom* or a happy union of strength of body and resolution, or firmness of mind, sufficient to have resisted attacks of the French at the Battle of Waterloo.^27^

The cult of fortitude in Waterloo surgery often focused on the role of national character, although, at this point in time, seldom of race in any ‘scientific’ sense. Eighteenth-century ‘cabinet wars’ were widely understood as rational acts of self-assertion by states, not necessarily as ‘patriotic’ struggles, even if national feelings played a part. Although the war against France was finally won by essentially *ancien régime* armies, in the wake of the French *levée en masse*, there was also an increasing sense of war as a conflict between peoples.^28^ The memory of Waterloo reflected this shift, with views of the battle often emphasising the role of national character in determining victory and defeat, even though the battlefield was remarkably polyglot. For example, the English memoirist Charlotte Eaton, who was nearby in Brussels during the campaign, described the victory in starkly nationalist terms. She expressed full confidence that, ‘leagued with foreigners who could not be depended upon, and with allies who had been defeated … under every disadvantage British valour would still be triumphant, as it had ever been in every contest and at every period’.^29^

The standard narrative of Waterloo, that it was the result of British endurance in the face of French attack, played to well-known clichés of national traits. In his 1862 novel *Les Misérables*, Victor Hugo (whose father was a Napoleonic general) included a long section on Waterloo. Eager not to give too much credit to Wellington, he ascribed the victory to a fickle deity and to the national virtues, the ‘obstinate coolness … the ancient classic courage … the English firmness, the English resolution, the English blood’ of the British soldiers, contrasted with the ‘superhuman instinct, a flaming glance’, of the French. As he put it, ‘The iron soldier is worth as much as the Iron Duke’.^30^ This view of the battle may have been flawed, especially since only around a third of Wellington’s army was British (the rest being Dutch, Belgian and German, even before one counts Blücher’s Prussian force), but it was profoundly reassuring to British audiences.^31^ Discussions of surgery at Waterloo reflected such notions of ‘natural’ British resilience, courage and calm. For instance, the Exeter surgeon John Haddy James was impressed by the ‘silent heroism’ he saw during the ‘grim’ scenes of medical treatment in the wake of the battle, especially of British troops. He wrote that, ‘When one considers the hasty surgery performed on such an occasion, the awful sights the men are witness to, knowing that their turn on that blood-soaked operating table is next, seeing the agony of an amputation however swiftly performed, and the longer torture of a probing, then one realizes fully of what our soldiers are made’.^32^ Similarly, Edward (Ned) Costello, an Irish NCO in the Rifle Brigade who lost a finger in the Waterloo campaign, praised the bravery and ‘the cool, unflinching spirit of a British soldier’ during ‘a surgical operation’.^33^

One of the oldest tropes in the historiography of war is building up the strength of your enemies so as to magnify one’s achievement. It is thus no surprise to see many British sources praising the capacity of French troops to endure surgery.^34^ For instance, Charles Bell praised their fortitude of the ‘formidable’ French patients, even if he also believed them to be nothing but a ‘race of trained banditti’.^35^ As Bell reported, the French after Waterloo were capable of singing under appalling circumstances:

These fellows are brought from the field after lying many days on the ground, many dying, many in agony, many miserably racked with pain and spasms, and the fellow next to him mimics him and gives it a tune. ‘Ah, ha! vous chantez bien!’^36^

A number of stories about Frenchmen exhibiting bravery in surgery became standard anecdotes, reflecting supposed undying loyalty to Napoleon and French verve rather than the stolid virtues ascribed to their British counterparts. For instance, tales of one who ‘tossed his amputated arm in the air, with a feeble shout of “vive l’Empereur” and another wounded Frenchmen who cried out last words that he would ‘cheerfully shed the last drop in his veins for the great Napoleon!’ and a third who said ‘An inch deeper and you’ll find the Emperor’ as a bullet was removed from his chest, were often repeated.^37^

Ned Costello expressed a more sceptical view about the fortitude of French soldiers compared to their British opponents. Having seen a lot of ‘cutting off legs and arms’ in Brussels after Waterloo, he wrote about an ‘incident’ that ‘may in some measure show the difference of the two nations’. He described an ‘English soldier’ undergoing the amputation of an arm while holding ‘the injured limb with his other hand without betraying the slightest emotion, save occasionally helping out his pain by spitting forth the proceeds of a large plug of tobacco, which he chewed most unmercifully while under the operation’. In contrast, a nearby Frenchman whose shoulder was being probed by a surgeon looking for a ball was ‘bellowing lustily’. The noise caused by the French soldier so ‘annoyed’ the British soldier that ‘as soon as his arm was amputated, he struck the Frenchman a smart blow across the breech with the severed limb, holding it at the hand-wrist, saying, ‘Here take that, and stuff it down your throat, and stop your damned bellowing!’^38^ While other Irishmen of his generation had very different loyalties, Costello evidently found satisfaction in an anecdote that seemed to affirm the image of a stout British soldier, unabashed by a mere amputation, demonstrating his physical and moral superiority over a defeated Frenchman.

Over the past few years there has been a trend in historiography towards emphasising the range of the emotional repertoire of the nineteenth-century British soldier, what Holly Furneaux (writing primarily about the Crimean War) has called the British ‘military man of feeling’.^39^ On one level, the tales of glorification of British composure in surgery could seem to flatly contradict that approach, reflecting instead the traditional clichés of the stiff upper lip that that scholarship has questioned. It is certainly the case that the reality and subsequent depictions of Britons under the knife at Waterloo provided a good deal of ‘raw material’ for the developing ideology of British emotional restraint. On the other hand, the cult of surgical fortitude was also intimately connected to the culture of sensibility, since an interest in the feelings of soldiers was in some ways a prerequisite for a celebration of their fortitude. As Furneaux has argued, British military culture in this period had a strong narratological commitment to heroic tropes of mercy and stoicism rather than to an explicit approval of violence per se. Medical scenes of pain, compassion and patriotic endurance could thus be framed not as a matter of a lack of feeling but of overcoming fear and pain in an affective regime of manly self-possession.

The emotional world of Napoleonic-era surgery and its memory included intense ideas of blood sacrifice, one that reinforced British collective identity and that justified British power.^40^ The very fact that it was so bloody, in terms of both deaths and surgical operations, gave the battle more symbolic potency than a tactical victory of manoeuvre could ever have had. Some participants in the battle were explicit in linking British triumph to sacrificed bodies in surgery. An anonymous soldier described what happened to him at Waterloo when ‘A ball passed through my left thigh, upon which, like Jacob, I shall go halting to my grave, while a sabre-stroke, from a French soldier, inflicted such a wound on my arm, that amputation was indispensable’. Though he believed that politicians in post-war Britain were running the country in a way that was ‘manifestly wrong’, he continued to see his surgical experience in 1815 as a righteous sacrifice in the national cause

After serving my country during the flower of my days, after spilling my blood in her cause, and losing a portion of my animal self to secure or defend her honors [sic], I have been discharged from the service with the remuneration of a lieutenant’s pension. I reproach not my country, however; I love, I venerate every spot of British ground. Her laws and constitution are unrivalled by any nation beneath the sun.^41^

This conception of the battle shocked many, but can often be seen in implicit terms, for instance in Wordsworth’s poem *Thanksgiving Ode*, written for the first anniversary of Waterloo. Notoriously, it contains the line, ‘Yea, carnage is Thy daughter’, seeming to revel in the hurt done to human flesh at the battle.^42^ Tales of stoicism in surgery from the battle were significant not just as a sign of the emotional and moral character of the individuals concerned but of national emotional strength and power bought with sacrifice. The agonies of surgery, like the toll of dead, were part of the real and symbolic ‘butcher’s bill’ of blood spilled for glory – what Byron, whose cousin died at Waterloo, called ‘the red rain’ that ‘hath made the harvest grow!’ in *Childe Harold’s Pilgrimage.* Other, less prominent, poets wrote about the blood ritual element at Waterloo in less critical terms. Victorian versifier Edmund White’s *A Soldier at Waterloo* depicted a soldier in despair over the moral crime of killing his fellow man, before he is redeemed by a divine vision of the battle as a blood sacrifice and he kisses his own blood-stained sword.^43^ Looking back from 1900, Rudyard Kipling, in his *Song of the Dead* made this point very succinctly – ‘If blood be the price of admiralty,/Lord God, we ha’ bought it fair!’^44^ One could see the apparently happy unanaethesised surgical patient as representing a willing sacrificial victim in an assertion of group cohesion. The heavy use of the language of blood sacrifice in the French Revolution (for instance in the *Marseillaise*’s lines ‘Let an impure blood/Water our furrows’) meant that British observers generally avoided overt references of that kind, but at an implicit level, an almost Jacobin conception of blood, purity and nation was a vital element in the ideology of spiritualised warfare that underlay Romantic Militarism in Britain, too.

## Class and fortitude in Waterloo surgery

One of the principal themes in stories of composure in surgery at Waterloo was the sense that the increasingly respectable common soldier was capable, even enthusiastic, about enduring torments in his country’s cause.^45^ The construction of the memory of Waterloo involved an emphasis on the supposed unity of officers and men in a spirit of patriotic sacrifice.^46^ Wellington himself referred to the British Army as something alien to the national spirit, ‘a new force’ that ‘has arisen out of the extraordinary exigencies of modern times’, and, famously, referred to his troops as the ‘scum of the earth’.^47^ Whig ideology had always had a soft spot for the navy, if not for sailors, but feared the army as a potential tool for tyrants. However, victories and the advent of an era of greater mass political mobilisation meant that the image of the steadfast, loyal and brave private soldier came to have more influence than ever before, as was reflected in the booming genre of military memoirs by normal soldiers.^48^ This incorporation of the common soldier into the National Romance required both amnesia about scenes of murder and rape such as happened at Ciudad Rodrigo and a focus precisely on things like fortitude in surgery.^49^

Evidence from first-hand testimony does suggest that some common soldiers did show remarkable composure in surgery. For instance, in his memoir written in the 1840s, Thomas Jackson, a sergeant from the Coldstream Guards, provided a detailed account of the loss of his leg in an amputation while a prisoner during the campaign a year before Waterloo. In it, he wrote:

They had got me fixed upon the end of a long barrack-room table … a bason [sic] was brought for me to drink out of. I said, ‘Sir, let me have a good draught’. He poured out nearly a pint [of red wine], which I eagerly drank off. In an instant it raised up my spirits to an invincible courage. ‘Now, gentlemen’, said I, ‘go on, if you please’. The serjeant [sic] was preparing to blindfold me ‘Oh no,’ I said, ‘I shall sit still and see as well as the rest’. One of the surgeons sat on a stool, to hold the leg steady: the second ripped up my trousers and took down the stocking low enough, then he waited on the head surgeon … the tourniquet being placed painfully tight above the knee, he put his hand under the calf of the leg and setting the edge of the knife on the shin bone, at one heavy, quick stroke, drew it round till it met the shin bone again. All eyes were fixed on me, but I was too high spirited then to give way. The blood quickly following the knife, spread around and formed like a red fan, downwards, at the sight of which, the frightened, but sympathising Dutch lookers on, screamed as though they were being cut. They pitied me very much and said ‘poor sergean [sic] major’. … When the saw was applied, I found it extremely painful; it was worn out … it stuck as a bad saw would when sawing a green stick. I said, ‘Oh, Sir, have you not a better saw?’ He said he was sorry he had not, as they were all worn out. The bone got through, the next thing to be done was still more painful – that of tying up the ligatures: then followed the drawing down of the flesh to cover the end of the bone, and tightly strapped there with strips of sticking plaster; after this, strongly bandaged; thus ended the operation, which lasted about half an hour. It was curious enough. These gentlemen, finding me not squeamish, amused themselves by engaging my attention in a conversation.^50^

The way that the passage above combines a description of his terror (needing a stiff drink beforehand and the frank acknowledgement of the pain) and his bravado and fortitude (insisting on watching the operation and chatting away to the surgeons) fits with a broader pattern in eyewitness sources that suggests that masculine self-esteem generally played a greater role than any ideological considerations. Other common soldiers expressed outright scepticism about those ideological aspects. Having seen punishment floggings, Thomas Morris, a sergeant in the 73rd Regiment of Foot, felt ‘somewhat less disposed to hazard life and limb, in a service, with the probability of being repaid with such gross inhumanity and ingratitude’.^51^ Nevertheless, his sense of pride and no doubt an understanding of what it meant to experience real injury and be sent to army surgeons led him to veto one of his men from getting a medal because he remembered him running away to Brussels pretending to have been injured, with his arm in a sling.^52^ Whatever the reality of men’s experience in the battle itself, the memory of ordinary soldiers from the Waterloo period laid the foundation for a broader reach for patriotic martial spirit.^53^ The interest in other ranks is sometimes depicted as in a sense ‘democratic’, but the popular militarism of the post-Waterloo era provides very little evidence for support for mass participation in politics. Instead, the ordinary soldier was included into an ideology that was not *ancien régime* in character, but a reflection of the development of modern conservativism, in which the masses were to be brought in to uphold the status quo. As Linda Colley noted, ‘Waterloo made the world safe for gentlemen again’.^54^

While common soldiers were the object of increased interest, it was the composure and character of gentlemen officers that remained at the heart of the discourse of heroic fortitude under the knife. At a time when the officer corps remained socially exclusive and commissions were still purchased, the British officer was increasingly being portrayed as a ‘moral leader’ in terms of his manly stoicism.^55^ Anecdotes of surgical fortitude among officers often came with explicit references to the courage and decorum expected of members of that class. Joseph Thackwell, an officer of the 15th Hussars who lost an arm at Waterloo, wrote an account of his experiences that reflects the high degree of self-consciousness about calm in surgery among officers. He starts by nonchalantly mentioning that the weather was ‘very fine’, before going on to explain that his ‘very stiff and painful’ arm was amputated ‘close to the shoulder’ at 8:30. He acknowledges that the pain was ‘great, but asserts that he ‘bore it with fortitude, and was greatly complimented on my heroism’.^56^

Famous officers at Waterloo provided many of the most important anecdotes of surgical heroism, some of which became fixtures in myths of Romantic Militarism and the British National Romance. Indeed, the number of such cases gives one a sense of the scale of the surgical carnage involved. Thomas Picton, the most senior officer to die at Waterloo, had, it seems, sworn a surgeon to secrecy after being injured in the days before the battle in order to lead his troops, supposedly saying, ‘I give you my word of honour, both as a gentleman and an officer, that if you place my name in your report as unfit for duty, I will shoot you with my own hand’.^57^ Henry Hardinge, later Governor-General of India, missed most of the battle after having his left hand shattered by a shot. A later account of his courage in *The Wellingtonian* noted that he was unable to find a surgeon, so ‘he made a common soldier cut it off with his sabre’, which he bore ‘without flinching’, but also mentioning that he was ‘peculiarly sensitive, and could not bear the sight of blood’, and that he fainted at the sight of the hand the next day.^58^ Fitzroy Somerset (later Lord Raglan of Crimea fame) also underwent an amputation at Waterloo. An article from the 1860s described Somerset as ‘patient as an Indian’, submitting to the amputation ‘without a murmur’. It was as his severed limb was being taken away that Somerset uttered the line that ensured that the story would be remembered, calling after the attendant, ‘Hallo! Don’t carry away that arm till I have taken off my ring!’ It was the engagement ring his wife had given him.^59^

The powers of endurance of Henry Paget (then Earl of Uxbridge, later Marquess of Anglesey) at Waterloo provided the most well-known instance of hyperbolic courage in surgery, becoming the centre of a cult with its own shrine and relics. The oft-repeated anecdote which suggested that he responded to a cannon ball taking his leg off by saying ‘By God, I’ve lost my leg’, only to hear the Duke of Wellington reply, ‘By God, Sir, so you have’, matched a need for exemplary tales of aristocratic phlegm.^60^ Sometimes, such as in the 1970 blockbuster film *Waterloo*, these words are depicted in terms of pathos and brotherly feeling. Other commentators have seen Wellington’s response as deliberately cold and sarcastic since relations between the men were bad, after Paget, a married father of eight, had run off with Wellington’s brother’s wife in one of the era’s biggest scandals.

The best source we have for the events involved, the medical notes of the surgeon, John Hume, however, gives a rather different account of what happened.^61^ Hume’s notes show that the leg was not lost in an instant, but amputated.^62^ The editor of Hume’s notes was surely right to argue that, while the reality might not be as ‘picturesque’ at the myth, it did reflect a similar ‘degree of coolness, fortitude and endurance in a situation of most intense agony’. Hume was delayed from reaching him and when he arrived to see the wound, he was amazed by the state of mind of his patient. After Hume and his colleagues had explained that the leg would have to be removed, Uxbridge responded with equal calm, saying, ‘very well I am ready’. During the gruesome business of cutting off the leg, an assistant responsible for holding the leg moved so that the saw became stuck in the femur. Hume at first did not understand the problem and angrily said, ‘Damn the saw’. This prompted Uxbridge’s only words of the operation, ‘What is the matter?’, said with a smile, it seems. Otherwise he ‘neither uttered groan or complaint nor gave any sign of impatience or uneasiness’ throughout the amputation. Afterwards Hume found his skin ‘perfectly cool’ and his pulse only 66 beats per minute, and was certain that Uxbridge was ‘so far … from exhibiting any symptoms of what he had undergone in his countenance that I am quite certain had anyone entered the room they would have enquired of him where the wounded man was’.^63^

The whole business of Uxbridge’s leg and his coolness in surgery soon became legendary, with the story evolving and gaining dubious additions. The author of *Flying Sketches of the Battle of Waterloo* written ‘on the spot’ in 1815, reports being told ‘little anecdotes’ shortly afterwards, one of which involved being told that an observer ‘had seen Lord Uxbridge’s leg amputated, and that this nobleman not only bore the operation like a hero’.^64^ William Whitehead’s 1820 *A Poem of the Battle of Waterloo* included a note on ‘the heroic Earl of Uxbridge’ to the effect that, ‘During the amputation of his leg not a groan escaped from him, nor did a contortion of countenance indicate that he was undergoing the operation; but with a firmness equal to that which he displayed on the field, even while the instrument of the surgeon in this painful duty, he magnanimously exclaimed, “Who would not lose a leg for such a victory!”‘^65^ A footnote in John Booth’s *The Battle of Waterloo* included a longer version of this anecdote, having Uxbridge follow that line with ‘It is true, I have a limb less; but I have a higher name in the eyes of my country’.^66^ Later descriptions of Uxbridge’s amputation generally avoided discussing the grimness of the scene. Like the Belgian artist Constantius Fidelio Coene’s painting of the ‘imaginary’ meeting between Uxbridge and the Duke of Wellington after the amputation, they portrayed the amputation as something serene, clean, white and beautiful, not as the scene of horror that it must have been.^67^

The earl’s leg became arguably the most famous surgical relic of the whole Napoleonic Wars – part of the substantial material culture of the memory of the battle.^68^ ‘Visit the smiling plains of Waterloo when you will’, wrote the English journalist Joachim Hayward Stocqueler in 1857, ‘the indefatigable guide is at your side’ to show the tourist not only Hougemont and ‘the spots where Ponsonby and Picton fell’ but also where ‘Anglesey and Fitzroy Somerset lost limbs’.^69^ Robert Southey’s *The Poet’s Pilgrimage to Waterloo* (1816) included a description of how Uxbridge’s leg had been made part of the sightseeing offering.^70^ The Duke of Wellington himself described taking George IV to see the shrine to Uxbridge’s leg, whereupon, the King, who had generally been drunk and hungover on his European tour, ‘burst into tears’ – neat encapsulation of the compatibility of the cult of surgical fortitude and the cult of sensibility.^71^

Not everyone was impressed by the adoration of aristocratic sacrifice at Waterloo. William Makepeace Thackeray, whose novel *Vanity Fair* (1847) includes the most famous depiction of the aftermath of the battle, mocked the cult of Anglesea’s lost limb – ‘come, let us away and drop a tear over the Marquis[sic] of Anglesea’s leg!’ and expressed disgust at the fact that the British common soldiers’ names were not recorded on any memorial at the site.^72^ However, even Thackeray was sure that any Englishman would feel a patriotic ‘glow’ at the battlefield, and knew that his scepticism about aristocratic heroism in surgery went against the grain of British feeling.^73^ As Europe moved gradually to explicitly national citizen armies, this kind of ideologically loaded praise of the glory of the toleration of pain was almost as common as that celebrating élan. Stories of officers and men seemingly united in overcoming surgical pain at Waterloo left the Victorian period a potent legacy of tying the common soldier to the national cause and a veneration of chivalric officers.

## Conclusion

Along with Trafalgar, Waterloo remains arguably the most symbolically significant triumphs in the history of British arms, ‘the bedrock of Victorian self-confidence, pride and patriotism’.^74^ It was widely seen as a reward for national virtues and a validation of British power. Crucially, one of the chief virtues concerned was the ability to endure pain, including surgical pain. During the nineteenth century, the idea that Britons could ‘take it’ would develop in more overtly racialised terms as a rationale for empire. Even today, these victories continue to have great importance. Rory Muir is surely also right to suggest that triumph at Waterloo even today underpins ‘a belief that if necessary Britain could defy the rest of Europe united against her’, a point which perhaps explains some of our twenty-first-century predicaments.^75^

The reality of surgery at Waterloo does appear to have included stunning examples of composure, no doubt from complex motives related to ideas of masculine honour, regimental pride and national feeling. These were often exaggerated, simplified and channelled into familiar tropes and, joined by more or less invented stories, then turned into a key aspect of the British cultural memory of the wars. Shorn of the trauma of experience, stories of men enduring surgical agony, willing to literally sacrifice their blood and limbs in surgery as much as in combat, were a perfect fit for the developing culture of Romantic Militarism, new ideas of masculinity and to the British National Romance, giving a touch of steel and blood to the military man of feeling.^76^ In later military memoirs, as well as in literary, historical and medical texts, the memory of surgical heroism offered scope for the enjoyment of military victory within a ‘pleasure culture of war’ without contemplating the way that the battle also involved the imposition of death and dismemberment by the same British heroes.^77^ Portrayals of wounded bodies can serve many purposes, from terror tactics to crush opposition to anti-war messages, but the simplest way for a state to deal with the problem of surgical pain in ideological terms is to keep it out of sight. The discourse on heroic surgery at Waterloo is thus conspicuous for the way it acknowledged the suffering involved, but it did so not to lament it, but to celebrate it in a vision that praised superhuman composure or evoked a sentimental sympathy that asserted of the innocence of the observer.^78^

Despite examples of actual heroic composure recorded by witnesses, the romantic blood sacrifice myth-making that dominated later discourse on the subject in many ways did therefore distort the true nature of what happened. Wellington, perhaps not a likely candidate for questioning the idea of the glory of war, had fewer illusions about the reality of the battle than some later anecdote-mongers. The rest of his career would be based on the victory at Waterloo, but his response to the battle was couched not in terms of romance but of trauma:

My heart is broken by the terrible loss I have sustained in my old friends and companions, and my poor soldiers. Believe me, nothing excepting a battle lost, can be half so melancholy as a battle won … to win such a battle as this of Waterloo, at the expense of so many gallant friends, could only be termed a heavy misfortune, but for the result to the public.^79^

Any discussion of the dramatic or comical semi-mythological heroic discourse on Napoleonic-era surgery as a field of glory should keep one eye on its foundations in the actuality of killing and maiming in war.

## Footnotes

1 Stendhal, *The Red and the Black* (London: Kegan, Paul, Trench and Co., 1916), 6, 23.

2 Ibid.

3 Ibid.

4 Ibid.

5 Nancy Rosenblum, ‘Romantic Militarism’, *Journal of the History of Ideas,* 43/2 (1982), 249–68.

6 Yuval Noah Harari, *The Ultimate Experience: Battlefield Revelations and the Making of Modern War Culture, 1450*–*2000* (Basingstoke: Palgrave, 2008).

7 Holly Furneaux, *Military Men of Feeling* (Oxford: Oxford University Press, 2016), 2.

8 Michael Brown and Joanna Begiato, *Martial Masculinities: Experiencing and Imagining the Military in the Long Nineteenth Century* (Manchester: Manchester University Press, 2019); J. A. Mangan and Callum McKenzie, *Militarism, Hunting, Imperialism: ‘Blooding’ the Martial Male* (London: Routledge, 2010); A. Mangan ed. *Making Imperial Mentalities: Socialisation and British Imperialism* (Manchester: Manchester University Press, 1990); Graham Dawson, *Soldier Heroes and Adventure Narratives* (London: Routledge, 1994); Joanna Bourke, *Dismembering the Male* (London: Reaktion, 1999); Holly Furneaux, *Military Men of Feeling* (Oxford: Oxford University Press, 2016); John Tosh, *A Man’s Place: Masculinity and the Middle Class Home* (New Haven: Yale University Press, 2007),

9 Linda Colley, *Britons: Forging the Nation, 1707*–*1837* (New Haven: Yale University Press, 1992).

10 One exception to this relates to the great Napoleonic surgeon Dominique Jean Larrey, who suggested that British resilience was based on their ‘lymphatic and little irritable constitution’ and a high protein meat diet. J. Henry Dible, *Napoleon’s Surgeon* (London: William Heinemann, 1970), 305.

11 K. Watson, ‘Bonfires, Bells and Bayonets: British Popular Memory and the Napoleonic Wars’, in *War: Identities in Conflict, 1300*–*2000*, ed. by Bertrand Taithe and Tim Thornton (Stroud: Sutton, 1998), 95–112.

12 Dawson; Michael Paris, *Warrior Nation: Images of War in British Popular Culture* (London: Reaktion, 2000).

13 The English surgeon Haddy James thought casualties amounted to about 15,000 of Wellington’s men and 30,000 Frenchmen. Jane Vansittart, ed. by *Surgeon James’s Journal* (London: Cassell, 1964), 38.

14 Philip Shaw, *Waterloo and the Romantic Imagination* (Basingstoke: Palgrave, 2002), 9–19.

15 Ibid., 25, see also 9–29. As Shaw has pointed out, beyond Goya and Charles Bell, there are few depictions of wounded soldiers from the Napoleonic period that show an interest in the reality of suffering rather than in evoking a sentimental or vigorously patriotic mood: Philip Shaw, ‘Wars of Seeing: Suffering and Sentiment in Joseph Wright’s *The Dead Soldier,’* in *Soldiering in Britain and Ireland*, 89.

16 Henry Addison, *A Rough Sketch of the Field of Waterloo* (Brussels: Hauman and Co., 1839), 74.

17 William Hay, *Reminiscences 1808*–*1815 under Wellington* (Solihull: Helion and Company, 2017), 116.

18 Stendhal, *The Charterhouse of Parma* (Harmondsworth: Penguin, 1918), 84.

19 A Young Traveller, *Flying Sketches of the Battle of Waterloo* (London: For Private Circulaton, 1852), 28–9.

20 Michael Brown, ‘Redeeming Mr Sawbone: Compassion and Care in the Cultures of Nineteenth-Century Surgery’, *Journal of Compassionate Health Care* 4/13 (2017), 1–7.

21 Charles Bell, *Letters of Sir Charles Bell* (London: John Murray, 1870), 248. See Michael J. Aminoff, *Sir Charles Bell* (Oxford: Oxford University Press, 2017), 57–76; Philip Shaw, *Suffering and Sentiment in Romantic Military Art* (Farnham: Ashgate, 2013), 207; Thomas Dixon, *Weeping Britannia: Portrait of a Nation in Tears* (Oxford: Oxford University Press), 131.

22 Bell, 247.

23 Shaw, *Suffering and Sentiment*, 207.

24 For examples of scholarship on war and memory: David Blight, *Race and Reunion: The Civil War in American Memory* (Cambridge, MA; Belknap, 2002); Paul Fussell, *The Great War and Modern Memory* (New York: Oxford University Press, 2000); Jay Winter, *Sites of Memory, Sites of Mourning* (Cambridge: Cambridge University Press, 1998).

25 There is a substantial, fascinating and useful literature on Napoleonic-era military surgery, but it has not always been at the forefront of changing historiographical approaches to developments in surgery. Michael Crumplin, *Men of Steel: Surgery in the Napoleonic Wars* (Shrewsbury: Quiller Press, 2007); Michael Crumplin, *Guthrie’s War*: *A Surgeon of the Peninsula and Waterloo* (Barnsley: Pen and Sword, 2010); Anthony Brett-James, *Life in Wellington’s Army* (London: George Allen and Unwin, 1972), 250–70; Andrew Bamford, *Sickness, Suffering and the Sword: The British Regiment on Campaign, 1808*–*1815* (Norman, OK: University of Oklahoma Press, 2013); Martin Howard, *Wellington’s Doctors: The British Army Medical Services in the Napoleonic Wars* (Staplehurst: Spellmount, 2002), 125–54; M.H. Kaufman, *The Regius Chair of Military Surgery in the University of Edinburgh, 1806*–*55* (Amsterdam: Rodopi, 2003); Geoffrey L. Hudson, *British Military and Naval Medicine, 1600*–*1830* (Amsterdam: Rodopi, 2007); Richard L. Blanco, ‘The Development of British Military Medicine, 1793–1814’, *Military Affairs* 38/1 (1974), 4–10; J. M. Matheson, ‘Comments on the Medical Aspect of the Battle of Waterloo, 1815’, *Medical History* 10/2 (1966), 204–7; Toby Gelfand, *Professionalizing Modern Medicine: Paris Surgeons and Medical Science in the 18th Century* (Westport: Greenwood Press, 1980); Martine Devillers-Argouarc’h, *Charles Bell, Chirugien à Waterloo* (Paris: Michalon, 2015).

26 Horace Walpole, for instance, wrote in the 1780s that he feared the country would ‘remain dissipated till totally impoverished’ after losing the War of American Independence: Paget Toynbee, ed. by *The Letters of Horace Walpole* 16 vols. (Oxford: Clarendon Press, 1905), volume 13, 140.

27 Walter Scott, ‘Account of the Defence of Hougemont’, in *The Miscellaneous Prose Works of Sir Walter Scott* volume 1 (Edinburgh: Robert Cadell, 1816), 82.

28 Daniel Moran and Arthur Waldron, ed. by *The People in Arms: Military Myth and National Mobilization since the French Revolution* (Cambridge: Cambridge University Press, 2006); Mark Hewitson, *Absolute War: Violence and Warfare in the German Lands* (Oxford: Oxford University Press, 2017).

29 Charlotte Eaton, *Narrative of a Residence in Belgium during the Campaign of 1815* (London: John Murray, 1817), 102.

30 Victor Hugo, *Les Miserables* 3 vols. (London: Hurst and Blackett, 1862), volume 1, 289.

31 A brief comparison of the stolid virtues at Waterloo and the hyper-aggressive ‘zeal’ prized by the Royal Navy might suggest that together they do not amount to a coherent national style of war. While there is no doubt that many British units did display remarkable staying power during the battle, there were examples of some cracking under pressure: Rory Muir, *Wellington: Waterloo and the Fortunes of Peace 1815*–*1852* (New Haven: Yale University Press, 2015), 65–73.

32 Vansittart, ed. by 35–6.

33 Edward Costello, *The Adventures of a Soldier* (London: Henry Colburn, 1841), 294.

34 The Dutch troops at Waterloo were accused of cowardice or even of sympathy with the enemy, since many of them had been in the *Grand Armée*. Erwin Muilwijk, *Standing Firm at Waterloo* (London: Sovereign House, 2014), 144–5.

35 Bell, 241–2.

36 Ibid.

37 James Simpson, *A Visit to Flanders, in July 1815* (Edinburgh: William Blackwood, 1816), 23. See also Anon., *The Bloody Battle of Waterloo, and a Visit to the Field of Battle and the French Hospitals* (Falkirk, T. Johnston, 1822), 24; Anon., *Britain Triumphant on the Plains of Waterloo* (Burslem: John Tregortha, 1817), 193–4; Christopher Kelly, *The Memorable Battle of Waterloo* (London: Thomas Kelly, 1818), 98.

38 Costello, 294–5.

39 See the works by Furneaux and Dixon.

40 Douglas Hedley, *Sacrifice Imagined* (New York, Bloomsbury, 2011); Allen Frantzen, *Bloody Good: Chivalry, Sacrifice and the Great War* (Chicago University Press, 2004); Carolyn Marvin and David W. Ingle, *Blood Sacrifice and the Nation* (Cambridge: Cambridge University Press, 1999).

41 Anon., ‘The Disabled Soldier’, *Tales of the Wars: or, Naval and Military Chronicle,* 2 (8 July 1837), 224.

42 Philip Shaw, ‘Wordsworth, Waterloo and Sacrifice’, in Alex Houen and Jan-Melissa Schramm (eds), *Sacrifice and Modern War Literature* (Oxford: Oxford University Press, 2018), 20–33.

43 Edmund White, ‘A Soldier at Waterloo’, *Athelstan, a Tragedy: Life and Death, an Allegory and other Poems* (London: W. Strange, 1847), 161.

44 Rudyard Kipling, ‘The Seven Seas’, in *Stories and Poems* (Oxford: Oxford UP, 1999), 471.

45 Neil Ramsay, ‘“A Real English Soldier”: Suffering, Manliness and Class in the Mid-Nineteenth-Century Soldiers’ Tale’, in *Soldiering in Britain and Ireland, 1750*–*1850*, ed. by Catriona Kennedy and Matthew McCormack (Basingstoke: Palgrave, 2013), 136–58.

46 Linda Colley, ‘Whose Nation? Class and National Consciousness in Britain 1750–1830’, *Past and Present* 113/1 (1986), 97–117.

47 Quoted in Rory Muir, *Wellington: The Path to Victory, 1769*–*1814* (New Havem: Yale University Press, 2013), 228.

48 Neil Ramsay, *The Military Memoir and Romantic Military Culture, 1780*–*1835* (Farnham: Ashgate, 2011); J. E. Cookson, *The Armed Nation, 1793*–*1815* (Oxford: Clarendon, 1997).

49 Muir, *Wellington: The Path to Victory*, 442.

50 Thomas Jackson, *Narrative of the Eventful Life of Thomas Jackson* (Solihull: Helion, 2018), 77–8.

51 Thomas Morris, *Recollections of Military Service* (London: James Madden, 1845), 10.

52 Ibid., 290.

53 John Mackenzie, *Popular Imperialism and the Military* (Manchester: Manchester University Press, 1987).

54 Colley, *Britons*, 195.

55 Ramsay, ‘“A Real English Soldier”’, 150.

56 H.C. Wylly, *The Military Memoirs of Lieutenant General Sir Joseph Thackwell* (London: John Murray, 1908), 80.

57 Wellcome Library RAMC/922.

58 Anon., ‘Heroem Vitae’, *The Wellingtonian,* 2/15 (November 1872), 401.

59 ‘The Amenities of War’, *Hours at Home,* 6 (1867–8), 21.

60 John Hume, *Notes Waterloo* (Royal College of Surgeons, Edinburgh GD1/1/5).

61 Royal College of Surgeons, Edinburgh GD1/3/14.

62 John Hume, *Notes Waterloo* (Royal College of Surgeons, Edinburgh GD1/1/5).

63 Ibid.

64 A Young Traveller, 32.

65 William Whitehead, *A Poem of the Battle of Waterloo* (London: Thomas Davison, 1820), 67–8.

66 John Booth, ed. by *The Battle of Waterloo* (London: John Booth, 1817), 153.

67 Shaw, *Suffering and Sentiment*, 179–80.

68 The National Army Museum’s Waterloo 200 collection offers a fascinating glimpse into this material culture. Waterloo200.org.uk.

69 J. H. Stocqueler, *The British Soldier: An Anecdotal History of the British Army* (London: Wm S. Orr, 1857), 138. See also Marysa Demoor, ‘Waterloo as a Small “Realm of Memory”: British Writers, Tourism, and the Periodical Press’, *Victorian Periodicals Review* 48/4 (2015), 453–68.

70 Southey, *The Poet’s Pilgrimage to Waterloo* (London: Longman, Hurst, Rees, Orme and Brown, 1816), 212.

71 Muir, *Wellington: Waterloo and the Fortunes of Peace*, 80.

72 William Makepeace Thackerary, ‘Little Travels and Roadside Sketches’ in Anne Ritchie (ed.), *The Works of William Makepeace Thackeray* 13 vols. (New York: Harper Brothers, 1898), 298. I would like to express my gratitude to one of the anonymous reviewers of this article for *War & Society* for suggesting this addition to this article.

73 Ibid.

74 Muir, *Wellington: Waterloo and the Fortunes of Peace*, 584.

75 Ibid.

76 Catriona Kennedy, ‘John Bull into Battle: Military Masculinity and the British Army Officer during the Napoleonic Wars’ in *Gender, War and Politics: Transantlantic Perspectives 1775*–*1830*, ed. by Karen Hagemann, Gisela Mettele and Jane Rendell (Basingstoke: Palgrave, 2010).

77 See Dawson.

78 Philip Shaw, ‘Wars of Seeing: Suffering and Sentiment in Joseph Wright’s *The Dead Soldier’*, in *Soldiering in Britain and Ireland*, 89. See also Shaw, *Suffering and Sentiment*, 3; Susan Sontag, *Regarding the Pain of Others* (New York: Farrar, Straus and Giroux, 2013), 91.

79 John Timbs, ed. by *Wellingtonia: Anecdotes, Maxims and Characteristics of the Duke of Wellington* (London: Ingram, Cooke and Co., 1852), 57.

